# Percent Fat Mass Increases with Recovery, But Does Not Vary According to Dietary Therapy in Young Malian Children Treated for Moderate Acute Malnutrition

**DOI:** 10.1093/jn/nxz037

**Published:** 2019-04-09

**Authors:** Christine M McDonald, Robert S Ackatia-Armah, Seydou Doumbia, Roland Kupka, Christopher P Duggan, Kenneth H Brown

**Affiliations:** 1 Children's Hospital Oakland Research Institute, Oakland, CA; 2 Department of Nutrition, University of California, Davis, CA; 3 Department of Public Health, Faculty of Medicine, University of Bamako, Bamako, Mali; 4 Nutrition Section, UNICEF, New York, NY; 5 Division of Gastroenterology, Hepatology and Nutrition, Boston Children's Hospital, Boston, MA; 6 Departments of Nutrition, and Global Health and Population, Harvard TH Chan School of Public Health, Boston, MA; 7 Bill & Melinda Gates Foundation, Seattle, WA

**Keywords:** acute malnutrition, anthropometry, body composition, child growth, Mali, supplementary feeding

## Abstract

**Background:**

Moderate acute malnutrition (MAM) affects 34.1 million children globally. Treatment effectiveness is generally determined by the amount and rate of weight gain. Body composition (BC) assessment provides more detailed information on nutritional stores and the type of tissue accrual than traditional weight measurements alone.

**Objective:**

The aim of this study was to compare the change in percentage fat mass (%FM) and other BC parameters among young Malian children with MAM according to receipt of 1 of 4 dietary supplements, and recovery status at the end of the 12-wk intervention period.

**Methods:**

BC was assessed using the deuterium oxide dilution method in a subgroup of 286 children aged 6–35 mo who participated in a 12-wk community-based, cluster-randomized effectiveness trial of 4 dietary supplements for the treatment of MAM: *1*) lipid-based, ready-to-use supplementary food (RUSF); *2*) special corn–soy blend “plus plus” (CSB++); *3*) locally processed, fortified flour (MI); or *4*) locally milled flours plus oil, sugar, and micronutrient powder (LMF). Multivariate linear regression modeling was used to evaluate change in BC parameters by treatment group and recovery status.

**Results:**

Mean ± SD %FM at baseline was 28.6% ± 5.32%. Change in %FM did not vary between groups. Children who received RUSF vs. MI gained more (mean; 95% CI) weight (1.43; 1.13, 1.74 kg compared with 0.84; 0.66, 1.03 kg; *P* = 0.02), FM (0.70; 0.45, 0.96 kg compared with 0.20; 0.05, 0.36 kg; *P* = 0.01), and weight-for-length *z* score (1.23; 0.79, 1.54 compared with 0.49; 0.34, 0.71; *P* = 0.03). Children who recovered from MAM exhibited greater increases in all BC parameters, including %FM, than children who did not recover.

**Conclusions:**

In this study population, children had higher than expected %FM at baseline. There were no differences in %FM change between groups. International BC reference data are needed to assess the utility of BC assessment in community-based management of acute malnutrition programs. This trial was registered at clinicaltrials.gov as NCT01015950.

## Introduction

Globally, an estimated 50.5 million children <5 y of age suffer from acute malnutrition (AM) (1). This condition is characterized by wasting and can be further classified into moderate and severe forms according to the child's weight-for-height *z* score (WHZ), mid-upper arm circumference (MUAC), and/or the presence of bipedal edema (2). AM impairs immune function, heightens susceptibility to infection, and increases the risk of mortality (3, 4). Children with a WHZ < −3, and between −2 and −3 are at 9.4 and 3.0 times higher risk of death, respectively, than are children with a WHZ > −1 (5). The burden of AM is particularly high in the Sahel region of Africa. In 2013, an estimated 385,000 children <5 y of age were believed to be living with moderate acute malnutrition (MAM) in Mali (6).

Community-based management of acute malnutrition (CMAM) is a comprehensive system for the prevention, screening, diagnosis, and treatment of different forms of AM in a community setting (3). In Mali, children with MAM are typically treated as outpatients, using appropriate medicines and either processed food blends, such as super cereal plus, or dietary counseling on the use of locally available cereal–legume blends and a mixture of vitamins and minerals (7). However, lipid-based nutrient supplements (LNSs) and improved formulations of cereal–legume blends have recently been proposed as superior treatment approaches given their enhanced nutrient profile and ease of preparation (8).

In addition to the growing recognition that treatment strategies must be tailored to meet the specific nutritional needs of children with MAM (9), there is increasing awareness that multiple issues must be considered when determining whether a dietary intervention is effective. The child's amount and rate of weight gain or change in MUAC are often the only outcomes that are considered when evaluating whether an intervention has succeeded in treating a child with AM. However, weight gain alone does not necessarily imply an improvement in health status. The measurement of body composition (BC) is an important part of pediatric nutritional assessment because it provides more detailed information on a child's nutritional stores and the type of tissue that has been accrued. However, BC is seldom assessed in the context of CMAM programs. Furthermore, there have been few investigations on the effects of newly designed dietary products on the BC of children with MAM.

We previously reported the results of a cluster-randomized, community-based effectiveness trial of 4 different dietary supplements on growth and recovery from MAM among young Malian children (10). The objectives of the study were to compare the change in percentage fat mass (%FM) and other BC parameters according to the type of dietary supplement received, and recovery status at the end of the 12-wk intervention period in a subgroup of trial participants.

## Methods

### Study design and participants

The study (Management of Children with Moderate Acute Malnutrition in Mali—NCT01015950) was designed as a cluster-randomized, community-based effectiveness trial. Given the different physical characteristics of the 4 supplements, the trial was not able to be masked. Detailed methods of the trial have been published previously (10). Briefly, the parent study was conducted in 12 of the 20 community health centers (CSComs) in the health district of Dioila, which is ∼170 km southeast of Bamako, Mali. Average daily high temperatures in this region typically range from 31°C in August to 40°C in April.

Although all children <5 y of age are eligible to receive treatment for AM in Mali, our study was restricted to children 6–35 mo of age, given the relatively higher prevalence of wasting in this subgroup and our desire to limit variability in the expected growth outcomes. Bimonthly community-based screenings were conducted between May, 2010 and May, 2011 to identify potential study participants. Children were identified as having MAM using 2 sets of criteria. The first set of criteria was based on the 2006 WHO Growth Standards: a weight-for-length *z* score (WLZ) < −2 and ≥ −3, or MUAC <12.5 cm and ≥11.5 cm (11). The second set of criteria was based on Mali's national norms at the time of the study: weight-for-length <80th percentile and ≥70th percentile of the National Center for Health Statistics median, or MUAC <12.0 and ≥11.0 cm (7). All children who met either set of criteria and did not have edema during the screening were referred to the CSCom for possible study enrollment. Children who were confirmed as fulfilling the eligibility criteria during the clinic-based assessment were invited to participate in the trial, and written informed consent was sought from the child's parent. Children with severe anemia (hemoglobin <50 g/L), severe acute malnutrition (SAM) [WLZ < −3 based on the 2006 WHO Growth Standards (4), weight-for-length <70th percentile based on the National Center for Health Statistics median, MUAC <11.0 cm], or other illnesses requiring inpatient treatment were referred for medical care and excluded from the study. We also excluded children with congenital abnormalities; underlying chronic diseases, including known HIV infection; or a history of allergy to peanuts or previous serious allergic reactions requiring emergency medical care. Long distances between CSComs, logistical and financial constraints, and the need to collect samples in the morning hours prevented us from conducting the BC substudy in all 12 CSComs. Therefore, we enrolled into the BC substudy a subsample of study children that were enrolled in the 8 CSComs nearest to the district hospital.

All 8 CSComs were stratified according to their distance from the main village of Dioila and population density, then randomly assigned to 1 of 4 dietary interventions within each stratum at the beginning of the study, and then were randomly reassigned, within stratum, to a different dietary group after the first 3 rounds of screening. All study children reporting to a particular CSCom received the same dietary supplement during each round.

### Study interventions

The 4 food supplements provided were: *1*) ready-to-use supplementary food (RUSF; supplied by Nutriset) called “Supplementary Plumpy” at the time of the study, but renamed to Plumpy'Sup since then; *2*) corn–soy blend “plus plus” (CSB++; supplied by the World Food Programme), a refined cereal–legume–milk blend containing dehulled soybean flour, maize flour, dried skimmed milk, sugar, soya oil, and a micronutrient premix that is designed for children with MAM and is very similar to a current World Food Programme product known as Super Cereal Plus; *3*) Misola (MI; supplied by Misola, Mali), which is a locally produced micronutrient-fortified cereal–legume blend that is less refined than CSB++ and contains 60% millet or maize flour, 20% soy flour, 10% peanut flour, micronutrient premix, and amylase powder; and *4*) a cereal–legume locally milled flours mix (LMF), also less refined than CSB++, which was provided along with vitamin A–fortified oil and multiple micronutrient powder sachets. All 4 dietary interventions were distributed to participants at enrollment and each follow-up visit throughout the 12-wk intervention period. The supplements were supplied in a quantity to provide each child with 500 kcal/d until the next scheduled visit. Caregivers were instructed to provide the supplement in addition to the child's usual home diet and not to share the supplement with other family members.

### Study procedures

In addition to receiving the dietary supplements, all study children were treated according to Mali's national CMAM protocol in place at the time of the study (7). All children received high-dose vitamin A supplementation if they had not received a vitamin A supplement in the previous 3 mo; anti-helminthic treatment; and specific ambulatory treatment, as required, for malaria or other acute infections.

Background information on sociodemographic characteristics was collected during the first patient encounter. At enrollment and at each of the scheduled follow-up visits at the CSCom (i.e., 1, 2, 3, 4, 6, 8, 10, and 12 wk after enrollment), the child's weight was measured to the nearest 20 g using the Seca 383 electronic baby and child scale (Seca), length was measured to the nearest 0.1 cm using a Shorr Board (Shorr Productions), and MUAC was measured to the nearest 0.1 cm with a Shakir Strip (UNICEF Supply Division). Length-for-age (LAZ), weight-for-age (WAZ), and WLZ were calculated using the 2006 WHO Growth Standards (12).

### BC assessment

The BC of subjects in the substudy was assessed at enrollment and at the end of the 12-wk intervention period using the deuterium oxide (^2^H_2_O) dilution technique. A baseline saliva sample of ≥2.5 mL was obtained from the child by holding cotton swabs in the mouth and using a syringe to express the absorbed saliva into two 3.6-mL Corning cryotubes. A preweighed dose of 4.00 g ^2^H_2_O, with an isotopic enrichment of 99.8% (Cambridge Isotope Laboratories), was then transferred from a vial to a syringe and administered orally to the child. To ensure that the entire dose of ^2^H_2_O was ingested by the child, the same syringe was used to rinse the vial twice with 1.5 mL of pure water, which was then administered to the child. Saliva samples were collected using the same procedure as described above 2.5 and 3.5 h after the ^2^H_2_O dose was administered. All saliva samples were temporarily stored in a portable refrigerated cooler and then transferred to a −20°C freezer at the end of the day.

Children were allowed to breastfeed and to consume water, bananas, and/or biscuits supplied by the study staff throughout the BC assessment procedure. The amount of breast milk consumed was estimated by weighing the child to the nearest 10 g with an electronic infant balance (Seca 354) immediately before and after the feeding. The mass of food or water consumed was estimated by weighing the amount served and the uneaten portion with an electronic balance with 0.1-g precision (MyWeigh Triton T2) and calculating the difference. The total water intake was then calculated by multiplying the mass of each food or liquid consumed by the water content of the particular food or liquid, as reported in the USDA Food Composition Database (13), and then summing all amounts and the amount of water used to rinse the ^2^H_2_O vial.

The ^2^H_2_O enrichment of the duplicate saliva samples was analyzed using Fourier Transform Infrared Spectroscopy at St. John's Research Institute in Bangalore, India. The ^2^H_2_O concentration of the baseline predose saliva sample was subtracted from the postdose saliva sample that had the highest ^2^H_2_O concentration. Total body water (TBW) was calculated using the following equation:

(1) \begin{eqnarray*}
{\rm{TBW}} &=& [{(^2}{{\rm{H}}_2}{\rm{O}}\,{\rm{dose}}{/^2}{{\rm{H}}_2}{\rm{O}}\,{\rm{enrichment)}} \times 900 \times 0.96 \times 0.944]\nonumber\\
&& -\, {\rm{water}}\,{\rm{intake}}\,({\rm{L}}),
\end{eqnarray*}

where 900 represents the molecular weight of ^2^H_2_O relative to water, 0.96 represents the nonaqueous hydrogen exchange, and 0.944 represents the estimated fractionation of the isotope (14). Fat-free mass (FFM) was calculated by dividing TBW by the proportion of water in FFM, which we obtained from age- and sex-specific reference data (15). Fat mass (FM) was then calculated as the difference between body weight and FFM. FFM index (FFMI) and FM index (FMI) were also calculated by dividing FFM and FM, respectively, by length squared. The percentage of body weight as fat mass (%FM) was also calculated.

### Sample size

We estimated that a sample size of 102 subjects per group was required to detect an effect size of 0.5 SD units in the difference in the change in FFM from baseline to 12 wk between groups, assuming a type I error rate of 0.05, power of 80%, and 15% attrition rate. Based on results from 2 previous studies of BC in young children in low-income countries, we expected the specified effect size to translate to a difference in the change in FFM of 0.17–0.28 kg. This effect size also corresponded to a difference in the change in %FM of ∼3.1 percentage points between groups (16, 17).

### Statistical analysis

All subjects with plausible BC data at both the enrolment visit and the 12-wk visit were included in the analysis. Descriptive statistics were used to summarize maternal, socioeconomic, and child characteristics according to treatment group at baseline. The mean ± SD was reported for all BC and anthropometric measures at baseline and the Tukey–Kramer test was used to detect any differences between groups. Paired *t* tests were used to identify any differences in mean values for the BC measures at baseline compared with 12 wk among all study subjects.

We used generalized linear models to estimate the adjusted mean change in all BC and anthropometric measures from baseline to the end of the 12-wk intervention period, by study group. The Tukey–Kramer test was used post hoc to identify any significant differences in the adjusted means between groups. We also used mixed linear regression models to compare the mean change in all BC and anthropometric measures, adjusted for child age, sex, weight at baseline, and value of the outcome variable at baseline, between children who had recovered from MAM at the end of the 12-wk intervention period and those who had not recovered. In these models, “recovery” was defined as a WHZ ≥ −2 and a MUAC ≥12.5 cm. Finally, we conducted correlation analyses and paired *t* tests to compare BC parameters among a subgroup of children who had ^2^H_2_O and bioelectrical impedance analysis (BIA) measurements available.

All statistical analyses were performed using SAS version 9.2 (SAS Institute Inc.).

### Ethics

The study protocol was approved by the Nutrition Division of the Malian Ministry of Health and the Ethical Review Committees of the University of Bamako Faculty of Medicine; University of California, Davis; and Boston University.

## Results

**FIGURE 1:**
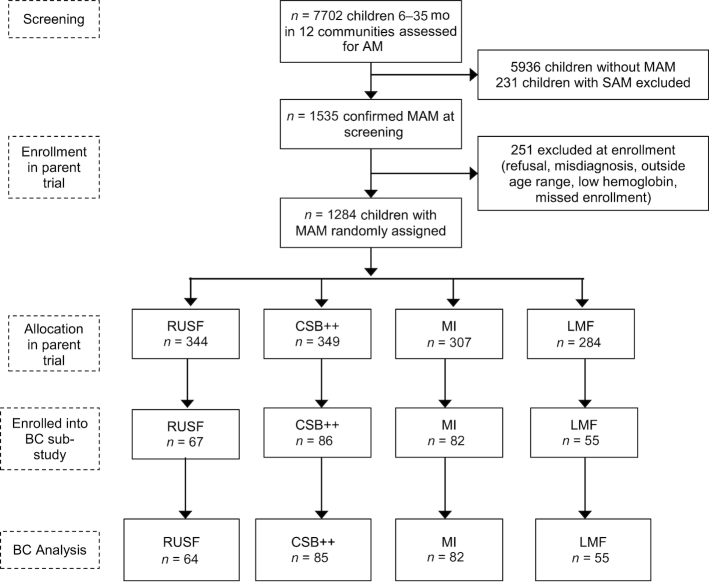
Profile for the BC substudy. AM, acute malnutrition; BC, body composition; CSB++, corn–soy blend “plus-plus”; LMF, locally milled flours mix; MAM, moderate acute malnutrition; MI, Misola; RUSF, ready-to-use supplementary food; SAM, severe acute malnutrition.

Of the 1284 children who were enrolled in the larger effectiveness trial, 290 children attending the 8 CSComs nearest to the district hospital were included in the BC substudy. This represented ∼38% of the total number of children who were enrolled in the main trial from these 8 CSComs. We excluded 1 child who did not have BC data available at both time points and 3 children who had a %FM >40%, which we deemed implausible and likely due to a methodological error in assessment. This left 286 children who were included in all analyses (**Figure 1**). As shown in **Table 1**, the vast majority of mothers in all 4 groups were married housewives with no formal education. The size of an average household exceeded 12 people, and most households used a bicycle as their primary mode of transportation. Mean child age at random assignment was ∼14.7 mo and just less than half of all participants were male. Mean child MUAC and WLZ at baseline ranged from 11.9 to 12.2 cm and from −2.11 to −2.23, respectively, across the 4 intervention groups. The distribution of most baseline characteristics did not significantly vary between children who were enrolled in the BC substudy and those who were not. However, children enrolled in the BC substudy had a slightly lower proportion of married mothers (96.5% compared with 98.6%; *P* = 0.02), their households owned fewer cows/goats on average (14.1 ± 20.1 compared with 23.3 ± 46.7; *P* = 0.003), and they exhibited higher mean LAZ and WAZ scores at baseline (–2.16 ± 1.19 compared with –2.40 ± 1.30; *P* = 0.006, and –2.76 ± 0.77 compared with –2.90 ± 0.86; *P* = 0.02, respectively).

**TABLE 1:** Characteristics of study participants at baseline, by treatment group^1^

	RUSF (*n* = 64)	CSB++ (*n* = 85)	Misola (*n* = 82)	LMF (*n* = 55)
Maternal characteristics				
No formal education	59 (93.7)	72 (84.7)	66 (80.5)	49 (89.1)
Can read or write	4 (6.1)	12 (14.1)	12 (14.6)	7 (12.7)
Profession				
Housewife	56 (88.9)	57 (67.1)	58 (70.7)	45 (81.8)
Agriculture	3 (4.8)	25 (29.4)	19 (23.2)	8 (14.6)
Other	4 (6.4)	3 (3.5)	5 (6.1)	2 (3.6)
Married	63 (98.4)	81 (95.3)	81 (98.8)	51 (92.7)
Household socioeconomic characteristics				
Number of people in the household/compound^2^	14.6 ± 9.6	13.9 ± 9.2	14.0 ± 8.2	12.7 ± 16.1
Number of cows/goats owned	6 [3, 13]	9 [3, 20]	8 [3, 16]	10 [5, 16]
Owns a radio	56 (88.9)	79 (92.9)	68 (82.9)	52 (94.6)
Mode of transportation				
Walk	4 (6.4)	5 (6.0)	2 (2.4)	3 (5.8)
Bicycle	39 (61.9)	68 (81.0)	51 (62.2)	36 (69.2)
Motorcycle	17 (27.0)	11 (13.1)	27 (32.9)	12 (23.1)
Other	3 (4.8)	0 (0.0)	2 (2.4)	1 (1.9)
Minutes to primary water source	5 [3, 10]	5 [2, 10]	4 [2, 5]	5 [2, 10]
Child characteristics				
Age at random assignment, mo	14.5 ± 7.2	15.8 ± 7.9	13.8 ± 6.6	14.7 ± 6.6
Male sex	33 (51.6)	40 (47.1)	30 (36.6)	25 (45.5)
Weight, kg	6.93 ± 1.22	7.23 ± 1.24	6.82 ± 0.99	7.16 ± 0.95
Length, cm	70.5 ± 6.7	72.3 ± 7.0	70.1 ± 5.3	71.6 ± 5.7
Mid-upper arm circumference, cm	11.9 ± 0.5	12.2 ± 0.5	12.0 ± 0.5	12.0 ± 0.5
Weight-for-length *z* score	–2.20 ± 0.76	–2.23 ± 0.62	–2.23 ± 0.59	–2.11 ± 0.72
Length-for-age *z* score	–2.39 ± 1.30	–2.09 ± 1.18	–2.15 ± 1.07	–2.04 ± 1.25
Weight-for-age *z* score	–2.90 ± 0.85	–2.74 ± 0.72	–2.78 ± 0.73	–2.63 ± 0.80

1 Values are mean ± SD, *n* (%), or median [IQR]; *n* = 286, unless otherwise noted. CSB++, corn–soy blend “plus-plus”; LMF, locally milled flours mix; RUSF, ready-to-use supplementary food.

2 A household was defined as a group of people who are primarily dependent on an individual person, currently eat from the same pot, and live under the same roof or in the same compound.

**Table 2** shows the mean ± SD body weight, TBW, FM, %FM, MUAC, and WLZ among all subjects at baseline and at the end of the 12-wk intervention period. With the exception of %FM, each parameter significantly increased over time (*P* < 0.0001). FFM increased by a mean of 13.3% and FM increased by 17.1%. As shown in **Table 3**, there were significant differences in TBW, FFM, and MUAC between groups at baseline (*P* < 0.05). Multivariate analysis of change on the various BC parameters from baseline to 12 wk indicated significant differences in the amount of length, body weight, FM, FMI, WLZ, and MUAC gained between groups. Pairwise comparisons revealed that children in the RUSF group had greater gains in weight, FM, FMI, and WLZ than children in the MI group (1.43 compared with 0.84 kg, *P* = 0.02; 0.70 compared with 0.20 kg, *P* = 0.01; 0.89 compared with 0.10 kg/m^2^, *P* = 0.04; and 1.17 compared with 0.57, *P* = 0.03, respectively) and children in the LMF group demonstrated a greater increase in MUAC than children in the MI group (1.24 compared with 0.57 cm; *P* = 0.008). Finally, there was a nonsignificant trend towards increased length gain among children in the CSB++ group compared with the MI group (3.33 compared with 2.52 cm; *P* = 0.06).

**TABLE 2:** Body composition of all study participants at baseline and the end of the 12-wk intervention^1^

	Baseline	Final	Change	*P* ^2^
Length, cm	71.1 ± 6.3	73.9 ± 6.0	2.73 ± 1.12	<0.001
Body weight, kg	7.03 ± 1.13	8.05 ± 1.30	1.02 ± 0.53	<0.0001
Total body water, kg	3.96 ± 0.73	4.47 ± 0.74	0.51 ± 0.38	<0.0001
FFM, kg	5.03 ± 1.10	5.70 ± 0.99	0.69 ± 0.53	<0.0001
FFMI,^3^ kg/m^2^	9.88 ± 0.86	10.4 ± 0.86	0.53 ± 0.90	<0.0001
FM, kg	1.99 ± 0.42	2.33 ± 0.54	0.33 ± 0.50	<0.001
FMI,^4^ kg/m^2^	3.97 ± 0.80	4.27 ± 0.84	0.30 ± 0.93	<0.0001
FM, % of body weight	28.6 ± 5.32	29.0 ± 4.79	0.36 ± 6.6	0.31
Mid-upper arm circumference, cm	12.1 ± 0.51	12.9 ± 0.75	0.84 ± 0.72	<0.0001
Weight-for-length *z* score	–2.20 ± 0.66	–1.47 ± 0.83	0.73 ± 0.71	<0.0001

1 Values are mean ± SD, *n* = 286, unless otherwise noted. FFM, fat-free mass; FFMI, fat-free mass index; FM, fat mass; FMI, fat mass index.

2 Obtained from a paired *t* test.

3 FFMI was calculated as FFM (in kilograms) divided by length (in meters), squared.

4 FMI was calculated as FM (in kilograms) divided by length (in meters), squared.

**TABLE 3:** Effect of dietary regimen on change in length and body composition of study participants over the 12-wk intervention^1^

	RUSF (*n* = 64)	CSB++ (*n* = 85)	Misola (*n* = 82)	LMF (*n* = 85)	*P* ^2^
Baseline					
Length, cm	70.5 ± 6.7	72.3 ± 7.0	70.1 ± 5.3	71.6 ± 5.7	0.11
Body weight, kg	6.93 ± 1.23	7.23 ± 1.24	6.82 ± 0.99	7.16 ± 0.95	0.08
TBW, kg	3.85 ± 0.82	4.08 ± 0.77	3.82 ± 0.65	4.12 ± 0.74	0.02
FFM, kg	4.88 ± 0.99	5.18 ± 1.05	4.84 ± 0.87	5.23 ± 0.98	0.03
FFMI,^3^ kg/m^2^	9.82 ± 0.82	9.84 ± 0.75	9.78 ± 0.76	10.2 ± 1.13	0.06
FM, kg	2.01 ± 0.43	2.04 ± 0.40	1.97 ± 0.35	1.92 ± 0.51	0.40
FMI,^4^ kg/m^2^	4.08 ± 0.73	3.94 ± 0.76	4.04 ± 0.70	3.79 ± 1.05	0.21
FM, % of body weight	29.3 ± 4.9	28.5 ± 4.8	29.2 ± 4.7	27.2 ± 7.0	0.10
WLZ	–2.20 ± 0.76	–2.23 ± 0.62	–2.23 ± 0.59	–2.11 ± 0.72	0.74
MUAC, cm	11.9 ± 0.50^a^	12.2 ± 0.55^b^	12.0 ± 0.46^a,b^	12.0 ± 0.45^a,b^	0.009
Change from baseline to end of 12-wk intervention^5^					
Length, cm	3.38 (2.80, 3.96)	3.33 (2.81, 3.85)	2.52 (2.16, 2.88)	2.94 (2.50, 3.37)	0.009
Body weight, kg	1.43 (1.13, 1.74)^a^	1.03 (0.76, 1.30)^a,b^	0.84 (0.66, 1.03)^b^	1.24 (1.01, 1.46)^a,b^	0.02
TBW, kg	0.66 (0.45, 0.88)	0.59 (0.40, 0.78)	0.45 (0.32, 0.58)	0.56 (0.40, 0.72)	0.32
FFM, kg	0.87 (0.59, 1.14)	0.76 (0.52, 1.01)	0.59 (0.43, 0.76)	0.74 (0.54, 0.94)	0.35
FFMI,^3^ kg/m^2^	0.68 (0.22, 1.14)	0.45 (0.04, 0.86)	0.52 (0.24, 0.79)	0.55 (0.22, 0.89)	0.21
FM, kg	0.70 (0.45, 0.96)^a^	0.19 (–0.03, 0.41)^c^	0.20 (0.05, 0.36)^b,c^	0.52 (0.34, 0.71)^a,b^	0.009
FMI,^4^ kg/m^2^	0.89 (0.43, 1.34)^a^	–0.08 (–0.48, 0.32)^c^	0.10 (–0.17, 0.38)^b,c^	0.55 (0.22, 0.88)^a,b^	0.02
FM, % of body weight	2.88 (0.26, 5.50)	–1.13 (–3.44, 1.18)	–0.54 (–2.12, 1.04)	1.51 (–0.40, 3.41)	0.12
WLZ	1.23 (0.83, 1.63)^a^	0.54 (0.18, 0.90)^a,b^	0.49 (0.25, 0.74)^b^	0.99 (0.70, 1.28)^a,b^	0.02
MUAC, cm	1.17 (0.79, 1.54)^a,b^	0.87 (0.54, 1.21)^a,b^	0.57 (0.35, 0.80)^b^	1.24 (0.96, 1.51)^a^	0.01

1 Values are mean ± SD, *n* = 286, unless otherwise noted. Groups were compared using unadjusted general linear models. Means in a row without a common letter are significantly different (Tukey–Kramer test, *P* < 0.05). The only exception to this was the comparison in the mean change in FM between the RUSF and CSB++ groups, which had a *P* value of 0.051. CSB++, corn–soy blend “plus-plus”; FFM, fat-free mass; FFMI, fat-free mass index; FM, fat mass; FMI, fat mass index; LMF, locally milled flours mix; MUAC, mid-upper arm circumference; RUSF, ready-to-use supplementary food; TBW, total body water; WLZ, weight-for-length *z* score.

2 *P* value reflects the Type III sum of squares for the treatment group in the model.

3 FFMI was calculated as FFM (in kilograms) divided by length (in meters), squared.

4 FMI was calculated as FM (in kilograms) divided by length (in meters), squared.

5 Values are adjusted means (95% CI), *n* = 286. Adjusted means in a row without a common letter are significantly different (Tukey–Kramer test, *P* < 0.05). Groups were compared by using general linear models that adjusted for month of enrollment, cluster, age, sex, and value of the outcome variable at baseline.

Approximately two-thirds of children in the BC substudy recovered from MAM by the end of the 12-wk intervention (**Table 4**). Because “recovery” was defined by achieving a WLZ ≥ −2 and MUAC ≥12.5 cm, it is not surprising that gains in body weight, TBW, WLZ, and MUAC were all significantly greater among recovered than among nonrecovered children (*P* < 0.0001). However, the recovered children also exhibited greater gains in FFM, FFMI, FM, FMI, %FM, and length than did the non-recovered children (*P* < 0.0001). We also observed that the recovered children had greater FM (2.02 compared with 1.87 kg; *P* = 0.002), but not %FM, at baseline.

**TABLE 4:** Change in anthropometric status and body composition from baseline to the end of the 12-wk intervention according to recovery status at end of treatment^1^

	Recovered at end of treatment (*n* = 182)	Not recovered at end of treatment (*n* = 104)	*P* value
Length, cm	2.99 (2.84, 3.14)	2.31 (2.11, 2.51)	<0.0001
Body weight, kg	1.28 (1.22, 1.35)	0.59 (0.50, 0.67)	<0.0001
Total body water, kg	0.61 (0.56, 0.66)	0.33 (0.26, 0.40)	<0.0001
FFM, kg	0.80 (0.74, 0.87)	0.44 (0.36, 0.53)	<0.0001
FFMI,^2^ kg/m^2^	0.69 (0.58, 0.81)	0.27 (0.12, 0.41)	<0.0001
FM, kg	0.51 (0.45, 0.57)	0.05 (–0.02, 0.13)	<0.0001
FMI,^3^ kg/m^2^	0.57 (0.46, 0.68)	–0.13 (–0.27, 0.01)	<0.0001
FM, % of body weight	1.32 (0.65, 1.99)	–1.19 (–2.07, –0.32)	<0.0001
Weight-for-length *z* score	1.08 (1.00, 1.16)	0.15 (0.04, 0.56)	<0.0001
Weight-for-age *z* score	0.85 (0.79, 0.92)	0.07 (–0.02, 0.16)	<0.0001
Length-for-age *z* score	0.07 (0.01, 0.12)	–0.18 (–0.25, –0.11)	<0.0001
MUAC, cm	1.20 (1.13, 1.27)	0.27 (0.17, 0.37)	<0.0001

1 Values are mean (95% CI), *n* = 286. Change from baseline to final was compared according to recovery status using a mixed linear regression model that adjusted for the value of the outcome variable at baseline, age, sex, cluster, and month of enrollment. “Recovery” was defined as a weight-for-height *z* score ≥ −2 and a MUAC ≥12.5 cm at the end of the 12-wk dietary treatment. FFM, fat-free mass; FFMI, fat-free mass index; FM, fat mass; FMI, fat mass index; MUAC, mid-upper arm circumference.

2 FFMI was calculated as FFM (in kilograms) divided by length (in meters), squared.

3 FMI was calculated as FM (in kilograms) divided by length (in meters), squared.

Among 190 study participants who had both ^2^H_2_O and BIA measurements available, %FM values were correlated at both time points (baseline: *r* = 0.22, *P* = 0.002; 12 wk: *r* = 0.30, *P* < 0.0001). Although the mean %FM as measured by BIA was 1.3 percentage points lower than the mean %FM as measured by ^2^H_2_O, at baseline (*P* = 0.01), there were no significant differences in mean %FM at 12 wk.

## Discussion

In this study of 286 young Malian children with MAM treated for 12 wk, we observed a greater increase in body weight, FM, FMI, and WLZ among children who received daily supplementation with RUSF compared with MI, and a greater increase in MUAC among children who received LMF compared with MI. Although we did not identify any significant differences in the change in %FM between groups, children who recovered from MAM by the end of the intervention exhibited greater gains in all BC parameters, including %FM. Our study is one of the first to compare changes in BC as a response to dietary supplementation in children with MAM in a low-resource, community-based setting.

BC reference data are extremely limited for children <5 y of age, which makes it challenging to compare the BC of our study participants with that of healthy children (18). We observed that although the mean TBW, FM, and FFM among all study participants were expectedly lower than the corresponding means in a cohort of 76 healthy US children of comparable age, the mean %FM of 29% appears higher than reference mean values, which range from 24.5% in 18-mo-old boys to 27.6% in 12-mo-old girls (15). The same observation was made when our findings were compared with reference values calculated by Fomon et al. (19) in the early 1980s. The discrepancy in %FM became even more pronounced when we compared our data with recently published BC data from populations in other resource-limited settings (20–22). We were able to confirm that the relatively high %FM values observed in our study population were not likely due to any methodological errors by showing that the %FM measurements as determined by ^2^H_2_O were comparable with those obtained by BIA in a subgroup of 190 study participants. Furthermore, other investigators have confirmed that the ^2^H_2_O procedure is an appropriate methodology for the assessment of BC in children with MAM (23).

Our finding of a greater increase in body weight and WLZ among children who received RUSF extends the main finding from the parent study (10). In a recent trial involving children with MAM from Burkina Faso, Fabiansen et al. (20) also reported greater gains in body weight, WLZ, MUAC, and overall recovery rates among children who received LNS for 12 wk in comparison with CSB. However, in contrast to our findings, which illustrated a significantly greater increase in FM, independent of length accrual, among children who received RUSF compared with MI, the Burkinabe children receiving LNS exhibited a slight increase in FFMI in comparison with those receiving CSB. Furthermore, although the mean amount of weight gained over the 12-wk intervention period was similar in both studies (1.01 kg in our study compared with 0.90 kg in the Burkina study), the composition of the accrued mass was quite different. Of the mean mass accrued by children in our study, ∼31% was in the form of FM. This value is somewhat lower than the range of 40–46% of mass accrued as fat in Peruvian and Indian children recovering from SAM (24, 25). However, the corresponding proportion in the Burkinabe children with MAM was only 6.7%, which resembles the proportion of body weight accrued as fat during normal growth in well-nourished children (19). Fabiansen et al. (20) speculated that the relatively higher proportion of fat accrual in the older studies of children with SAM could be due to a diet that was inadequate in zinc and other micronutrients that are critical for growth and FFM accrual. Although all 4 dietary supplements in our study met WHO recommendations for the nutrient contents of rehabilitation diets (9, 26), it is possible that underlying infection could have diverted essential nutrients typically used for FFM synthesis and caused the subsequent deposition of excess energy as fat. Alternatively, the greater proportionate gain in FM by children recovering from SAM may be because they start out with lower %FM than those with MAM, especially when the diagnosis of SAM is based on the presence of edema. Although there was no association between %FM at baseline and recovery status in our study population, there was an inverse association between %FM at baseline and change in %FM over the intervention period. However, it is possible that the latter observation could reflect regression to the mean due to imprecision of the measurements.

Breastfeeding status, history of malnutrition, and zinc status have all been suggested to play a role in tissue accrual or BC status (27–29). Unfortunately, we did not have comprehensive data on these factors in our population, nor are they consistently reported in other study populations. It is also possible that the hydration coefficient of FFM in well-nourished children may not be valid for use in children with AM. Boutton et al. (27) have reported that the relatively high weight-for-height values observed in a group of stunted Peruvian children were not reflective of increased FM, but rather increased hydration of FFM in the form of extracellular water, in comparison with well-nourished children. Clearly, more research is needed to better understand the biological mechanisms governing BC and the partitioning of tissue accrual, as well as possible methodological factors that may explain the diversity in BC data from various populations.

The utility of BC assessment in the context of CMAM programs is difficult to assess based on the findings from our study. Based on previous studies involving children with AM (20, 22, 24, 25), we expected the %FM of this population of children with MAM to be lower than that of healthy children and that BC assessment would enable us to identify whether and how effectively supplementation restored “reference” BC as children recovered. Although we observed significant differences in all BC parameters according to recovery status at the end of the 12-wk intervention, our inability to detect any significant differences in %FM across intervention groups using the ^2^H_2_O dilution method indicates that BC measurements may not be sufficiently sensitive to compare the nutritional adequacy of different therapeutic diets even in a research setting.

Several strengths of our study should be noted. Despite its challenges, we assessed BC using the ^2^H_2_O dilution technique, which is considered the best available method for field use. We also implemented multivariate statistical modeling in our analysis of the data to account for the effects of important covariates such as age, sex, baseline BC, and seasonality. Furthermore, our study provides much-needed data from an understudied, but very important, population: young children with MAM. However, our study was not free from limitations. First, although our sample size of 286 is one of the largest to date, we did not achieve our goal of 102 children per group (i.e., 408 total), which reduced our ability to detect a possible small difference in %FM between groups. This was primarily due to the difficulty in obtaining adequate postdose saliva samples from young children living in an arid environment with extremely high temperatures. Second, logistical constraints prevented us from randomly selecting participants for this substudy from the larger parent trial, which resulted in some differences between groups at baseline and limits the generalizability of our findings. Children enrolled in the substudy attended the 8 CSComs that were closest to the district hospital and may have had better access to medical services. The mean LAZ and WAZ at baseline were also slightly higher among children enrolled in the BC substudy than among those who were not enrolled, indicating a better nutritional status at baseline. Third, we evaluated the change in BC from enrollment to the end of the 12-wk intervention period in all children. We did not evaluate the change in BC from enrollment to the actual time of recovery. This is an important distinction because supplementation would stop and children would “graduate” from the treatment program once their WLZ and MUAC surpassed thresholds of –2 and 12.5 cm, respectively, in an actual programmatic setting. This may have attenuated differences between groups. Finally, the diversity of the participants’ age at baseline (6–35 mo of age) and the corresponding variability in the expected BC trajectory over the 12-wk intervention period make it difficult to compare our findings with those of other studies.

In conclusion, supplementation with RUSF resulted in a greater increase in body weight, absolute FM, and WLZ among Malian children with MAM in comparison with a locally produced, less refined, micronutrient-fortified cereal–legume blend. Although no differences in %FM were observed between groups, children who recovered from MAM at the end of the 12-wk intervention did exhibit a larger increase in %FM, along with all other BC parameters than children who had not recovered. International BC reference data and further research on the physiological mechanisms underpinning BC changes in malnourished populations are urgently needed before the utility of BC assessment in CMAM programs can be comprehensively assessed.

## Notes

Supported by UNICEF Mali and UNICEF West and Central Africa Regional Office (to KHB), the World Food Programme (to KHB), the Goldman Fund (to KHB), and Helen Keller International (to KHB). Supported in part by NIH grants K24DK104676 and 2P30 DK040561 (to CPD).

Author disclosures: CMM, RSA-A, SD, RK, CPD, and KHB, no conflicts of interest.

The organizations who provided financial support for the study did not play any role in the design or implementation of the study, nor were they involved in the analysis or interpretation of the data. RK is a UNICEF staff member. The views expressed in this article are those of the author and may not reflect official UNICEF policies.

Abbreviations used: AM, acute malnutrition; BC, body composition; BIA, bioelectrical impedance analysis; CMAM, community-based management of acute malnutrition; CSB++, corn–soy blend “plus-plus”; CSCom, community health center; FFM, fat-free mass; FFMI, fat-free mass index; FM, fat mass; FMI, fat mass index; LAZ, length-for-age *z* score; LMF, locally milled flours; LNS, lipid-based nutrient supplement; MAM, moderate acute malnutrition; MI, Misola; MUAC, mid-upper arm circumference; RUSF, ready-to-use supplementary food; SAM, severe acute malnutrition; TBW, total body water; WAZ, weight-for-age *z* score; WHZ, weight-for-height *z* score; WLZ, weight-for-length *z* score; ^2^H_2_O, deuterium oxide; %FM, percentage fat mass.
